# SARS-CoV-2 Mutations Responsible for Immune Evasion Leading to Breakthrough Infection

**DOI:** 10.7759/cureus.29544

**Published:** 2022-09-24

**Authors:** Chetan Sahni, Priyoneel Basu Roy Chowdhury, Deepa Devadas, Ashish Ashish, Nitish K Singh, Abhay Yadav, Manpreet Kaur, Shivani Mishra, Shani Vishwakarma, Royana Singh

**Affiliations:** 1 Anatomy, Institute of Medical Sciences (IMS) Banaras Hindu University (BHU), Varanasi, IND; 2 Zoology, Kalinga Institute of Social Sciences Deemed University, Bhubaneswar, IND; 3 Multidisciplinary Research Unit/Anatomy, Institute of Medical Sciences (IMS) Banaras Hindu University (BHU), Varanasi, IND

**Keywords:** next genome sequencing, b.1.617.2 (delta-variant), breakthrough infection, immune evasion, sars-cov-2, covid-19 retro

## Abstract

Background and objectives: India had faced a devastating second outbreak of COVID-19 infection, in which a majority of the viral sequences were found to be of the B.1.617.2 lineage (Delta-variant). While India and the world focused on vaccination, reports of vaccine-immunity evasion by the virus, termed “breakthrough cases”, emerged worldwide. Our study was focused on the primary objective to identify the mutations associated with breakthrough infections SARS-CoV-2.

Methods: In our study, we extracted the SARS-CoV-2 RNA (ribonucleic acid) from reverse transcription-polymerase chain reaction (RT-PCR) positive COVID-19 patients, and 150 random samples were sent for sequencing to the Centre for Cellular & Molecular Biology, Hyderabad. Whole genome sequences of 150 SARS-CoV-2 viral samples were analyzed thoroughly. We mostly found B.1.617 and its sub-lineages in the genomic sequencing results.

Results and interpretation: On further analysis of patient data, it was seen that nine patients had been vaccinated against the SARS-CoV-2 previously. These nine patients had B.1.617/B.1 or A strains, and all of them had similar genomic variations in spike proteins as well as non-structural proteins (NSPs). The mutations seen in these sequences in the Spike (S), NSPs, and open reading frame (ORF) regions would have produced amino acid changes known to improve viral replication, confer drug resistance, influence host-cell interaction, and lead to antigenic drift.

Conclusions: Increased virulence culminating in vaccine immunity evasion may be inferred from these specific mutations. Our study adds to the growing body of evidence linking rapidly emerging mutations in the S (Spike) and ORF genes of the SARS-CoV-2 genome to immune evasion.

## Introduction

The SARS-CoV-2 pandemic, characterized by symptoms ranging from mild fever to respiratory distress, multi-organ failure, and death, has drastically changed public health paradigms worldwide. The second wave of the SARS-CoV-2 pandemic in India has seen the emergence of several variants, designated variants of concern [1]. While vaccination is underway on a massive scale, some reports have surfaced which indicate that the new variants may offer variations in antigenic presentation due to antigenic drift, resulting in altered antibody interaction, culminating in vaccine immunity evasion. The fully vaccinated (two doses) patients have also been re-infected (breakthrough cases) by the new variants worldwide [2,3]. Although this reduction in the efficacy of available vaccines has been observed more often with partial vaccination. Infections after the two weeks of vaccination were considered breakthrough infections.

The knowledge of genomic variations in the spike proteins and Non-structural proteins (NSPs) of SARS-CoV-2 is of utmost importance because the host cell invasion by the virus is mediated through the spike protein domain of the virus, which interacts with the host angiotensin-converting enzyme-2 (ACE-2) receptors to enable cellular entry. Once inside the host cells, the replication of the SARS-CoV-2 genome is dependent upon non-structural proteins coded by open reading frame (ORF), including chiefly NSP12, and a complex of NSP7 and NSP8, along with other NSPs, 15 in total. These proteins exist as oligomers, with the NSP7-NSP8 complex presenting itself as a hexadecamer (2X8). A monomer of NSP12 oligomerizes with one NSP7 and two NSP8 molecules, extending the RNA-binding surface. The two NSP8 molecules project helical N-terminal extensions, which interact with the newly synthesized RNA and its template, augmenting NSP-12 mediated replication [4-6].

The spike protein of the virus is of particular interest since its receptor binding domain (RBD) is a bipartite interface that interacts with the ACE2 receptor of the host [7]. Some of the mutations that have been recently identified in the spike protein of the virus include L452R and E484Q in the RBD. These mutations are associated with increased transmissibility and viral load, greater infectivity, and loss of neutralization sensitivity to vaccine-elicited sera, leading to increased virulence [8]. Another mutation, E484K, causes a 10-fold reduction in the effectiveness of neutralizing antibodies produced in response to vaccines [9].

These and other mutations in the ORF gene allow the newer variants to evade vaccine-induced immune responses and replicate faster [10]. A variant of concern discovered to be present in the majority of patients during the second wave of infections in India is B.1.167; including its sub-lineages; B.1.617.1 (S: T95I, G142D, E154K, L452R, E484Q, D614G, P681R, Q1071H), B.1.617.2 (Delta-variant) (S: T19R, G142D, 156del, 157del, R158G, L452R, T478K, D614G, P681R, D950N), and B.1.617.3 (S: T19R, L452R, E484Q, D614G, P681R). Of these, B.1.617.2, which is also known as the delta variant, has emerged as a more virulent version of the dreaded virus, prevalent in a large proportion of the patients found positive.

## Materials and methods

We isolated SARS-CoV-2 viral mRNA from nasopharyngeal and oropharyngeal samples collected from walk-in patients at the Sir Sunderlal Hospital, Banaras Hindu University, at the Multidisciplinary Research Unit, Banaras Hindu University, Varanasi. Of these, 150 mRNA samples were sent for whole genome sequencing to the Centre for Cell and Molecular Biology, Hyderabad. Subsequently, it was ascertained through the telephonic interview of all patients that nine of these patients had previously been vaccinated with a single or two doses, and hence represented cases of breakthrough infection. Then, we analyzed the mutations present in the viral mRNA extracted from all 150 samples retrospectively.

Patient identification and recruitment: Samples from any walk-in patient who wished to be tested for COVID-19 are accepted on a regular basis at the Out Patient Department, Room No. 103, Sir Sunderlal Hospital, Banaras Hindu University, Varanasi. These samples were received through proper channels and under adequate biosafety measures at the Multidisciplinary Research Unit, Institute of Medical Sciences, Banaras Hindu University, Varanasi. Vaccination status, symptoms, age, and gender were noted from patient records and re-verified through a personal telephonic interview by the clinical expert from our research lab. The information gathered was tabulated in Ms-Excel for graphical representation. Patient interviews were conducted after the patients had completed their home isolation period, and after obtaining informed consent, mostly over the telephone. The interviews were conducted using a semi-structured questionnaire by an experienced physician with a scientist to assist him. The information obtained was matched with patient records. The procedures involved were approved by the Institutional Ethical Committee vide letter no. Dean/2021/EC/2678.

Sample processing and RT-qPCR: The samples received were processed for lysis and mRNA extraction using magnetic bead separation (MagMax, Thermo Scientific, USA) and assayed for the presence of SARS-CoV-2 N/ORF1ab genes using Real-Time Reverse Transcription Polymerase Chain Reaction assay with human RNAse P as the internal control (BioRad CFX96, USA; COVISure real time PCR kit; Genetix Biotech Asia Pvt. Ltd., India).

Next generation sequencing: The mRNA extracted was sent with the maintenance of cold chain to the Centre of Cellular and Molecular Biology, Hyderabad, India, for whole genome next-generation sequencing via the IlluminaNovaseq 6000 platform at 500x to 4,000x read depth.

Viral genomic sequence analysis: The FASTA files uploaded to the GISAID database were downloaded, matched with existing laboratory records, and analyzed for the presence of amino acid changes via the COVSurver App (CoVsurver - CoronaVirus Surveillance Server; https://corona.bii.a-star.edu.sg ). We performed an in-silico analysis of the complete genomic sequences to predict functional impact of the mutations present in this variant on virus-host interactions in terms of viral transmissibility, virulence/lethality, and the immune escape. The properties conferred upon the virus by these amino acid changes were investigated via a keyword search on PubMed that included the notation of the amino acid mutation followed by the words “SARS-CoV-2”, “COVID19” and “viral oligomerization”, “antigenic drift”, and “vaccine evasion”.

## Results

**Figure 1:**
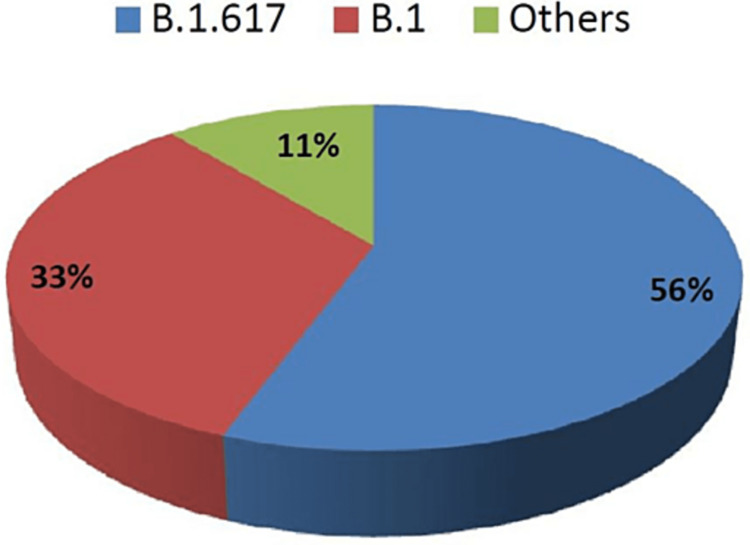
Percentage distribution of variants found in cases of breakthrough infection

Patients gladly volunteered details during the interview and reported being previously contacted over the telephone by government officials regarding their well-being during their home quarantine period. Interviews lasted 12±2 minutes on average. The information given regarding symptoms and contact or travel history is tallied with information previously on record. Vaccination history was taken twice to identify the breakthrough cases. We found 9 breakthrough cases out of 150 while taking the vaccination history. Vaccine breakthrough cases consisted mostly of young- to middle-aged males (41.67 ± 6.03 years). There was one case among these, male, who was 85 years of age. All of the cases had mild to moderate symptoms and were advised by the officials concerned to remain in home quarantine. The B.1.617 lineage was found to be predominantly present among these cases (5/9; with 4 cases of B.1.617.2 (Delta-variant) sub-lineage of B.1.617) (Figure 1).

**Figure 2:**
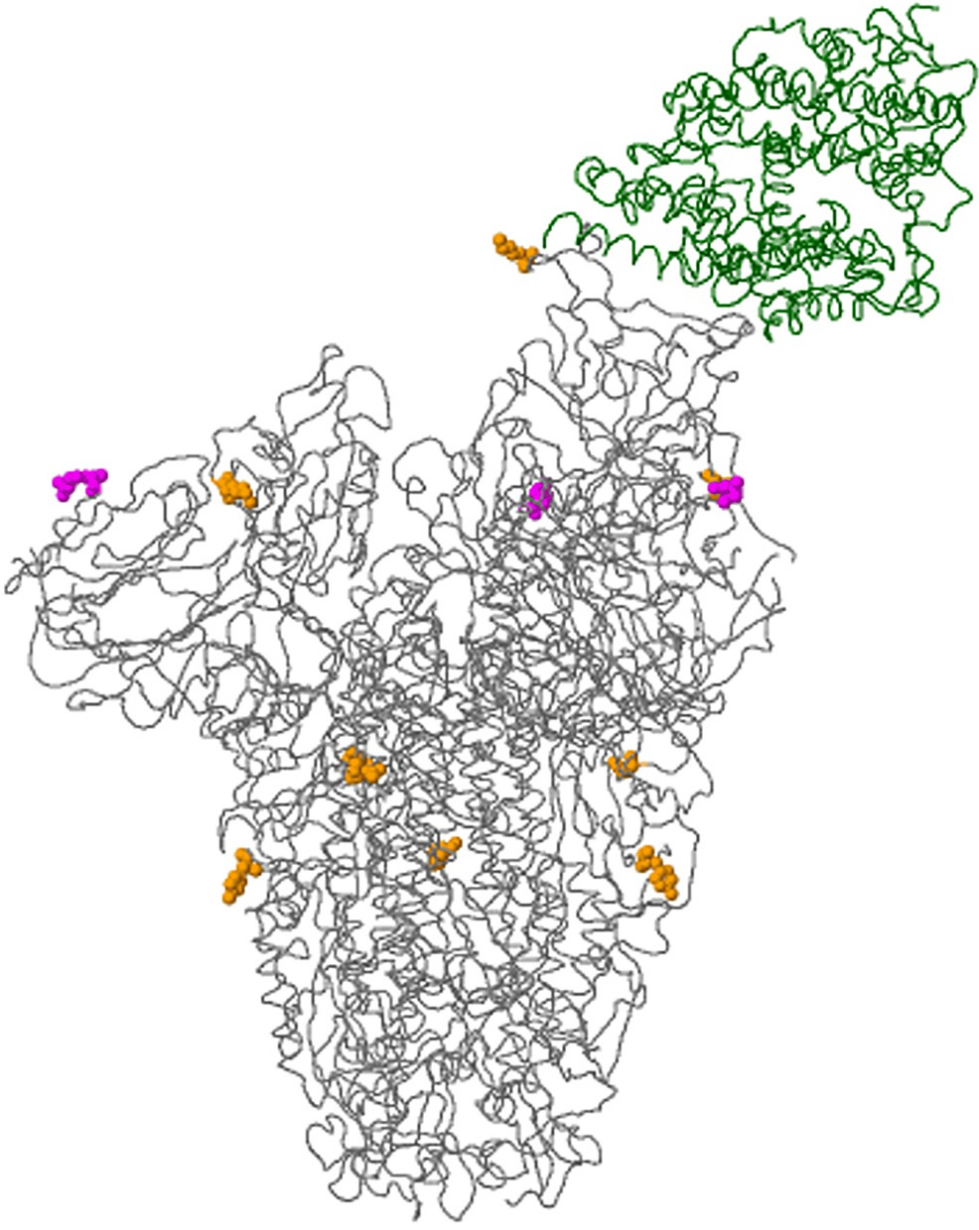
Representative 3D modelling of the Spike glycoprotein in interaction of human angiotensin converting enzyme-2 (ACE-2) receptor showing key amino acid substitutions in breakthrough case number EPI_ISL_2373554. Green part of this model is representing the host cell receptor, i.e., human ACE-2 (angiotensin converting enzyme-2) receptor, rest of the colorful globular structures representing the amino acid (aa) changes in spike protein (T19R(20) T478K D614G) of SARS-CoV-2. Data source: The figure has been generated from the genomic sequences of delta variant using CoVsurver app from Gisaid.org.

Apart from characteristic mutations of the spike protein known to occur in the B.1.617- lineages (Figure 2), there were also other changes in non-structural proteins that would improve viral oligomerization, thus favoring increased replication. We also found many amino acid changes in spike proteins and non-structural proteins were specifically present in the breakthrough infected patients. These are summarized in Table 1. In this table, along with the amino acid changes, accession id of all breakthrough cases is also mentioned. Which can be used to further analyze these mutations on covsurver-mutation-analysis-app (https://www.gisaid.org/epiflu-applications/covsurver-mutations-app/).

**Table 1:** Details of post-vaccination breakthrough cases, and amino acid changes found in the genomic sequence of SARS-CoV-2 in these cases. Marked Mutations (marked with *) are specific mutations, which we found associated with the breakthrough cases

Case NO.	Accession ID on GISAID	GENDER	AGE	RESULT (Pango lineage)/ CLADE	AA Changes at
1	EPI_ISL_2373512	FEMALE	32	B.1.617.2/G	Spike D614G, Spike D950N*, Spike L452R*, Spike P681R*, Spike T19R, M I82T, N D63G, N D377Y, N G215C, N R203M, NS3 S26L, NS7b T40I, NSP3 A488S, NSP3 G255V, NSP3 P1228L, NSP4 T492I, NSP4 V167L, NSP12 G671S, NSP12 P323L*, NSP13 P77L, NSP14 A394V
2	EPI_ISL_2373537	MALE	38	B.1.	Spike G1251V, NS3 T223I, NS3 V273M, NSP5 N65S, NSP6 T77A*
3	EPI_ISL_2400315	MALE	40	B.1.617.1/G	Spike P681R*, M I82T, NS3 S26L, NSP4 A286D, NSP6 F108L, NSP12 P323L, NSP13 M429I
4	EPI_ISL_2373545	MALE	29	B.1.617.2/G	Spike D614G, Spike D950N*, Spike L452R*, Spike P681R*, Spike T19R, Spike T478K, M I82T, N D63G, N D377Y, N L139F, N R203M, N R385K, NS3 G18V, NS3 S26L, NS3 V55F, NS7a L116F, NS7a T120I, NSP2 P129L, NSP3 P822L, NSP4 A446V, NSP6 V149A, NSP12 G671S, NSP12 P323L*, NSP13 P77L, NSP15 H234Y
5	EPI_ISL_2373546	MALE	30	B.1.617.2/G	Spike D614G, Spike D950N, Spike L452R*, Spike P681R*, Spike T19R, Spike T478K, M I82T, N D63G, N D377Y, N G215C, N R203M, NS3 S26L, NS7a T120I, NS7b T40I, NSP3 A488S, NSP3 P1228L, NSP3 P1469S, NSP4 T492I, NSP6 T77A, NSP12 G671S, NSP12 P323L*, NSP13 P77L, NSP14 A394V
6	EPI_ISL_2373554	MALE	85	B.1.617.2G	Spike D614G, Spike P681R*, Spike T19R, Spike T478K, M I82T, N D63G, N D377Y, N G215C, N R203M, NS3 S26L, NS7b T40I, NSP3 A488S, NSP3 P1228L, NSP3 P1469S, NSP4 T492I, NSP4 V167L, NSP6 T77A, NSP12 G671S, NSP12 P323L*, NSP13 P77L, NSP14 A394V
7	EPI_ISL_2373555	FEMALE	55	A/G	Spike D614G, M I82T, N D63G, NSP3 V765F, NSP6 T77A, NSP14 G189C
8	EPI_ISL_2373574	MALE	34	B.1/G	Spike D614G, NSP12 P323L*
9	EPI_ISL_2373609	MALE	32	B.1/G	Spike D614G, NSP12 P323L*

Case 1 was of a 32-year-old female, with no travel history. She was asymptomatic at the time of testing. The household of the patient had four members, all of whom were infected, with only the elderly reporting symptoms of fever. 

Case 2 was a male, 38 years old, with work-related travel history to an adjacent city. He reported being pre-diabetic and had had a high-grade fever during the course of the infection.

Case 3 was a 40-year-old male with a history of duty-related travel within his city and met “many” people. He reported recurrent fever and diarrhea, along with disturbances in sleep patterns.

Case 4 (29 years, male) was a frontline healthcare worker on active COVID19-related duty. He reported weakness and exhaustion as the only symptoms.

Case 5 was 30-year-old male who had no travel history, completed both rounds of vaccination two weeks before infection, and reported having fever, cough, a sore throat, and anosmia during the infection.

Case 6 was a cheerful 85-year-old male who had no travel history, was fully vaccinated, and had no noticeable symptoms other than a slight feeling of tiredness.

Case 7 was a female, 55 years old, with cough and anosmia, fully vaccinated, and had no travel history. She reported that others in her family had also been infected, but with mild symptoms, and had rapidly recovered.

Case 8 was a 34-year-old male who reported having fever for two weeks and feeling feverish for up to a month after infection. He reported having contact with patients at the workplace who had subsequently expired due to SARS-CoV-2-related complications. He had been partially vaccinated and attributed his survival to his vaccination status.

Case 9 was 32-year-old male, with no noticeable symptoms, no travel history, and fully vaccinated.

## Discussion

**Figure 3:**
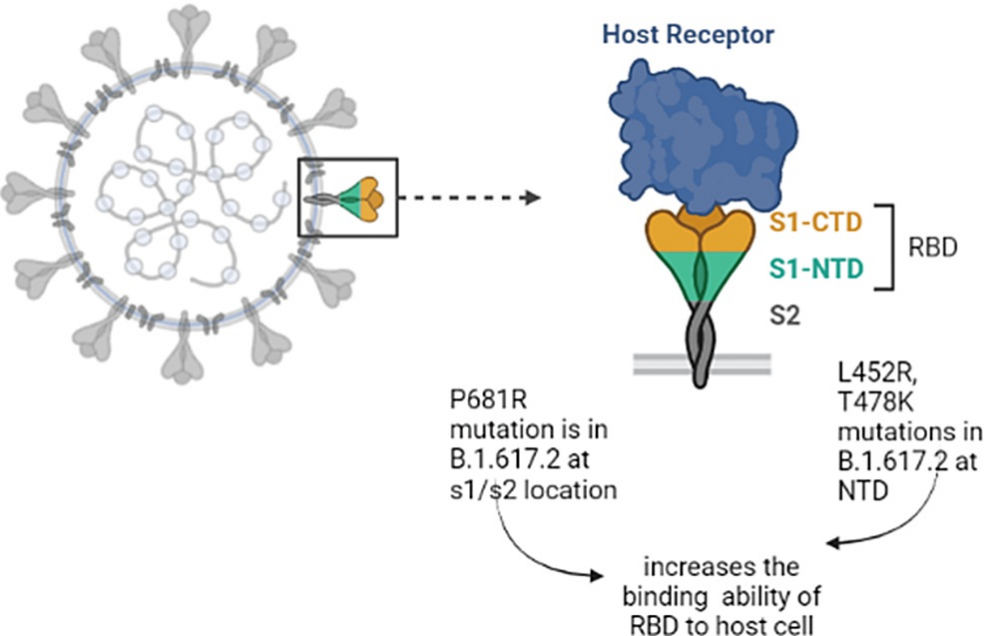
Schematic representing the effect of mutations in the spike protein of the SARS-CoV-2 virus on host-virus interactions The spike protein of SARS-CoV-2 has a receptor binding domain (RBD). This RBD has two protein units S1 & S2. The S1 unit has two domains, one is C-terminal domain (CTD) and other is N-terminal domain (NTD). (This Schematic diagram is the author's own creation.)

We found that different variants result in breakthrough infections, with the most vaccine evasion cases (4/9) found with B.1.617.2 (delta-variant) (Figure 3). Interestingly, one of the sequences of the B.1 variant that was associated with a partial breakthrough (only single-dose vaccine administered) case (Case 2) carried Spike G1251V, NS3 T223I, NS3 V273M, NSP5 N65S, and NSP6 T77A. The other B.1 variants we found in the samples isolated from unvaccinated cases (unpublished data) did not carry these mutations. Similarly, variant A that infected one of the breakthrough cases (Case 7) had amino acid changes N D63G, NSP3 V765F, NSP6 T77A, and NSP14 G189C. While Case 7 had mild symptoms, Case 2 was prediabetic and had moderate symptoms. NSP6 T77A was common in both these cases and did not occur in other A or B.1 variants that were found in the 145 samples from our laboratory.

The mutations found in the SARS-CoV-2 sequences in breakthrough patients infected with the B.1.617 lineage is similar to those found in samples from unvaccinated patients in our data set (unpublished data) except for some specific mutations, which were only present in breakthrough cases (enlisted in the table with * marked). The spike protein of SARS-CoV-2 has an RBD which helps the virus bind with the host cell receptor. This RBD has two protein units S1 & S2. The S1 unit has two domains, one is C-terminal domain (CTD) and the other is N-terminal domain (NTD) (Fig. 3). The variations (amino acid change) like P681R at S1-S2 location of the RBD and the amino acid changes like L452R & T478K increases the binding ability of the RBD to host cell receptors [11,12]. In the breakthrough cases, the samples which belong to B.1.617 lineage showed several common mutations in the Spike proteins like Spike D950N, Spike L452R, and Spike P681R which were reported in the literature to influence the Virulence, Host Change and Viral Oligomerization [7].

Symptoms in these patients ranged from mild to moderate, suggesting that vaccination may modulate the severity of symptoms, if not able to completely prevent infection. Travel history was not associated with infection, in five out of nine breakthrough cases that we studied. In retrospect, the infection at this stage would have been transmitted to sufficient members of the society to render travel history irrelevant as far as the strain of the virus was concerned. In these breakthrough cases, the ORF gene was also a hotspot of mutation in addition to the S gene, with amino acid changes signifying widespread changes in non-structural proteins (Table 1). NSP3 had the greatest number of amino acid changes in the breakthrough cases. This strongly suggests that viral oligomerization and, subsequently, its spread, would have been affected in the strains infecting these patients [10]. Amino acid changes occurring in these cases are known to not only improve viral replication but also confer drug resistance, influence host-cell protein/RNA interaction, increase virulence, and enable drug resistance [10]. Multiple changes in NSP3 (NSP3 P 822L, NSP3 V765F, NSP3 G255V, NSP3 A488S), as well as NSP4 T492I, NSP5 N65s, NSP12 P323L, and NSP15 H234Y, in combination, could have influenced viral oligomerization, modulating increased viral replication [4]. Additionally, NSP5 N65S confers increased virulence and drug resistance, while NSP3 P8221 and NSP3 V765F are predicted to affect host-virus interactions at the cell protein/ RNA level (GISAID COVSURVER mutations App, https://www.gisaid.org/epiflu-applications/covsurver-mutations-app/). NSP3 and NSP5 are responsible for proteolytic cleavage of the ORF polyprotein products into other NSPs responsible for the maintenance of the replication-transcription complex [4]. It is interesting to note that some of these amino acid changes are rarely found, and it is possible that the combination of these rare mutations adds to the virulence. The rate of the occurrence of mutations, and the multiple mutations might possibly indicate a rapidly changing and evolving virus undergoing selection for vaccine evasion in an increasingly vaccinated population.

This study has certain limitations. Since access to complete patient records is restricted due to privacy concerns, the specific progression of the fever, and the course of treatment apart from generic antibiotics and steroids, could not be known. Similarly, the study would have profited immensely from serological and immunological profiling of the patients to determine physiological status during infection by these variants, as well as antigenic interactions that would help understand the mode of vaccine evasion. However, the study serves to illustrate that mutational hotspots in certain genes may be consistently associated with immune evasion and vaccine breakthrough infections and that sequencing must be carried out in the course of routine surveillance during pandemics involving viruses.

## Conclusions

Our study adds to the growing body of evidence linking rapidly emerging mutations in the S and ORF genes of the SARS-CoV-2 genome to immune evasion. It is clear that in addition to B.1.617, B.1 and A strains can also evade vaccine-induced immune responses, even after two rounds of vaccination. Symptoms in these breakthrough infections patients ranged from mild to moderate, suggesting that vaccination may modulate the severity of symptoms, if not able to completely prevent infection. Added to the antigenic drift and altered interaction with antibody recognition sites seen in these variants, due to amino acid changes in the Spike protein, changes in the NSP group of proteins make the new variants a fearsome enemy, one that may evade vaccines, and thus leave the immune system defenseless. It is not currently understood how the patients themselves are different from those who received the vaccination, worked in similar settings, and were not infected. Two explanations present themselves. One could be that vaccinated candidates were exposed to an increased viral load due to the nature of their occupation, or that they could bear genetic susceptibility to the SARS-CoV-2 virus. The underlying host-virus interactions could be better understood by studying the exomes of the breakthrough cases to identify vulnerabilities. Thus, there is scope for further research along these lines.
