# Correction: SARS-CoV-2 Mutations Responsible for Immune Evasion Leading to Breakthrough Infection

**DOI:** 10.7759/cureus.c130

**Published:** 2023-08-07

**Authors:** Chetan Sahni, Priyoneel Basu Roy Chowdhury, Deepa Devadas, Ashish Ashish, Nitish K Singh, Abhay Yadav, Manpreet Kaur, Shivani Mishra, Shani Vishwakarma, Royana Singh

**Affiliations:** 1 Anatomy, Institute of Medical Sciences (IMS) Banaras Hindu University (BHU), Varanasi, IND; 2 Zoology, Kalinga Institute of Social Sciences Deemed University, Bhubaneswar, IND; 3 Multidisciplinary Research Unit/Anatomy, Institute of Medical Sciences (IMS) Banaras Hindu University (BHU), Varanasi, IND

This article has been corrected to include the following funding disclosure which was accidentally omitted by the submitting author at the time of submission:

"Funding required for this study was made available by Banaras Hindu University under a seed grant for new faculties under IoE to Dr. Chetan Sahni. The letter number is "R/Dev/D/IoE/Equipment/Seed Grant-II/2O22-23/48676", dated 07.06.2022."

The above statement is now visible in the 'Payment/services info' section of the Ethics Statement and Conflict of Interest Disclosures. The submitting author deeply regrets this omission.

